# Systems and synthetic biology-driven engineering of live bacterial therapeutics

**DOI:** 10.3389/fbioe.2023.1267378

**Published:** 2023-10-19

**Authors:** Kangsan Kim, Minjeong Kang, Byung-Kwan Cho

**Affiliations:** ^1^ Department of Biological Sciences, Korea Advanced Institute of Science and Technology, Daejeon, Republic of Korea; ^2^ KAIST Institute for the BioCentury, Korea Advanced Institute of Science and Technology, Daejeon, Republic of Korea; ^3^ Graduate School of Engineering Biology, Korea Advanced Institute of Science and Technology, Daejeon, Republic of Korea

**Keywords:** live biotherapeutics, probiotics, microbiome, synthetic biology, systems biology

## Abstract

The past decade has seen growing interest in bacterial engineering for therapeutically relevant applications. While early efforts focused on repurposing genetically tractable model strains, such as *Escherichia coli*, engineering gut commensals is gaining traction owing to their innate capacity to survive and stably propagate in the intestine for an extended duration. Although limited genetic tractability has been a major roadblock, recent advances in systems and synthetic biology have unlocked our ability to effectively harness native gut commensals for therapeutic and diagnostic purposes, ranging from the rational design of synthetic microbial consortia to the construction of synthetic cells that execute “sense-and-respond” logic operations that allow real-time detection and therapeutic payload delivery in response to specific signals in the intestine. In this review, we outline the current progress and latest updates on microbial therapeutics, with particular emphasis on gut commensal engineering driven by synthetic biology and systems understanding of their molecular phenotypes. Finally, the challenges and prospects of engineering gut commensals for therapeutic applications are discussed.

## 1 Introduction

The human intestine is densely populated with an estimated 10^13^–10^14^ native commensals and an exhaustive catalog of enzymes essential for host metabolism (Sender et al., 2016). The collective genetic pools of the gut microbiota are estimated to surpass those of humans by nearly three orders of magnitude (Tierney et al., 2019) and contribute as much as 10% of the metabolites in the host bloodstream (Wikoff et al., 2009). The gut microbiota cooperates to support important chemical reactions that are otherwise absent in host metabolism. For example, the saccharolytic species of *Bacteroides* harbor carbohydrate-active enzymes that catabolize complex glycans into simpler substrates and short-chain fatty acids, which serve as nutrients accessible to other commensals and the host (Luis et al., 2018). In addition, the conversion of primary bile acids into their secondary forms in mammals is mediated by microbiota-derived bile salt hydrolases, the lack of which interferes with cell cycle regulation, immunity, and insulin secretion (Collins et al., 2023).

Therefore, the human intestine represents an important interface of host-microbe interactions, which have broad implications in diverse facets of host biology, from simple nutrient metabolism to complex roles such as immunomodulation. Thus, any aberrant shift in the gut microbiota composition, termed dysbiosis, could inadvertently lead to disease; associations with infection (Buffie et al., 2015), gut inflammation (Franzosa et al., 2019), and metabolic disorders (Smith et al., 2013), among various others (Koeth et al., 2013) have been reported. With the increasing realization that host–microbe interactions play an integral role in host biology, many studies have focused on microbial therapeutics, a growing field that entails the use of engineered bacterial chassis or synthetic microbe consortia for disease treatment.

Traditional means of microbe-based therapeutic interventions largely entailed administration of individual microbial strains or their consortia that intrinsically harbor therapeutically-relevant properties (Schultz, 2008; Khan et al., 2022). The microbe-based therapeutic modalities are gaining more tractions thanks to recent advances in systems and synthetic biology, which enabled customization the microbial cells and synthetic consortia with an aim to address a range of diseases with a better efficiency, while minimizing the inherent limitations that undermine the utility microbial-based therapeutics. The rapid progress in the research and development of microbial therapeutics is exemplified by the rapid increase in the number of enrolments in clinical trials investigating microbiota-based therapeutics (Dronkers et al., 2020), with over 4,000 clinical trials using the term “gut microbiota” registered at ClinicalTrials.gov to date. Analytical tools that incorporate multi-omics analysis and *in silico* simulations allow for spatiotemporal analysis of host-microbe interactions at resolutions down to a single metabolite (Heinken et al., 2023). Importantly, the integrated systems analysis could facilitate elucidation of structural and functional roles of gut microbiome in association with disease manifestation, enabling exploration of potential targets for therapy. This effort could ultimately translate to rational design of natural or synthetic microbial consortia to reverse intestinal dysbiosis and its associated complications, as exemplified by the case studies of *Clostridioides difficile* infection (CDI) (Buffie et al., 2015). The emerging utility of synthetic microbial chassis in therapy is largely facilitated by modularized bioparts that enable the execution of user-programmed functions, such as site-specific therapeutic delivery, in a highly reproducible manner. In addition, expanding synthetic biology toolkits now cater to non-model gut microbes that were previously intractable to genetic engineering, providing ways to circumvent the inherent drawbacks associated with non-commensal model strains. In this review, we outline the latest advances in microbial therapeutics using systems and synthetic biology frameworks for novel discoveries and strain engineering, with an emphasis on the engineering of gut bacteria.

## 2 Systems biology approach to understanding microbe–host interactions

Decades of omics biology research on the human gut microbiome have uncovered the complex interplay of host–microbe interactions at the molecular level, allowing us to consider the implications of such dynamics on the health of the host. In the past decades, many efforts have been devoted to characterizing the composition and patterns of the gut microbiota that correlate with specific host status, such as diet and age (Claesson et al., 2012), obesity (Turnbaugh et al., 2009), and malnutrition (Smith et al., 2013). Considering the postulation that the gut microbiota contributes as much as one-tenth of the metabolites in the host bloodstream (Wikoff et al., 2009), high-throughput metabolomics have been actively used to elucidate the link between the gut microbiome and host phenotypes. Such endeavors have enabled the mapping of microbiota-derived biochemicals in association with a range of host physiologies, including disease manifestations (Franzosa et al., 2019), xenobiotic metabolism (Zimmermann et al., 2019), and behavioral changes (Dohnalova et al., 2022). Importantly, integrative analysis of systems biology datasets and the development of computational platforms that consolidate multiple datasets into coherent information have considerably expanded our capacity to investigate host-microbe interactions.

Integrative systems biology analysis that reconciles multi-omics datasets with *in silico* modeling frameworks serves as an effective approach for revealing the implicit dynamics of host-microbe interactions. Briefly, the collective understanding of microbial genetics, genomics, and biochemistry is put into a single computational framework, the genome-scale metabolic model (GEM), which is a mathematical framework featuring a matrix of biochemical reactions that allows the prediction of metabolic flux distribution using linear programming. The optimization technique, flux balance analysis (FBA), calculates the most feasible metabolic state that maximizes a given objective function such as biomass generation or target metabolite production (Orth et al., 2010; Kim et al., 2021). The GEM also contains gene-protein-reaction associations that can be extended to assimilate -omics datasets, such as metagenomic readouts on the strain-specific relative abundance of a microbiome community (Heinken et al., 2019), serving as a platform to integrate and analyze disparate datasets (Monk et al., 2017). In particular, the integrative analysis of host–microbe interactions provides important inferences on implicit factors such as profiles of metabolites that are mutually cross-fed or subject to competition between the host and microbes (Heinken et al., 2013), the weighted contribution of a specific microbiome to host metabolism (Heinken et al., 2019), and microbiome-specific drug catabolism that affects host drug response (Heinken et al., 2023), which can be extrapolated further for predictive modeling of host biological status (Yang et al., 2022). More importantly, the ability to identify causal factors linked to an observed outcome and their mechanisms can translate to pragmatic applications, such as novel therapeutic modalities for enteric infection and inflammation (Buffie et al., 2015; Zhu et al., 2018). The knowledge on metagenomics, genetics, and biochemical profiles accumulating over decades has culminated in the “big-data-based” platforms that allow scalable prediction and simulation of potential impact of microbial metabolism on host physiology. Previously, constraint-based metabolic models that predict optimal metabolic fluxes for a given objective function (Heinken et al., 2023) and homology-based predictions of metabolic gene clusters that provide inference on metabolic pathway abundance in a given metagenome pool have been used (Pascal Andreu et al., 2021; Pascal Andreu et al., 2023). However, because of their highly multifaceted nature, host–microbe interactions remain one of the most actively explored avenues in systems biology (The Integrative, 2019).

**FIGURE 1:**
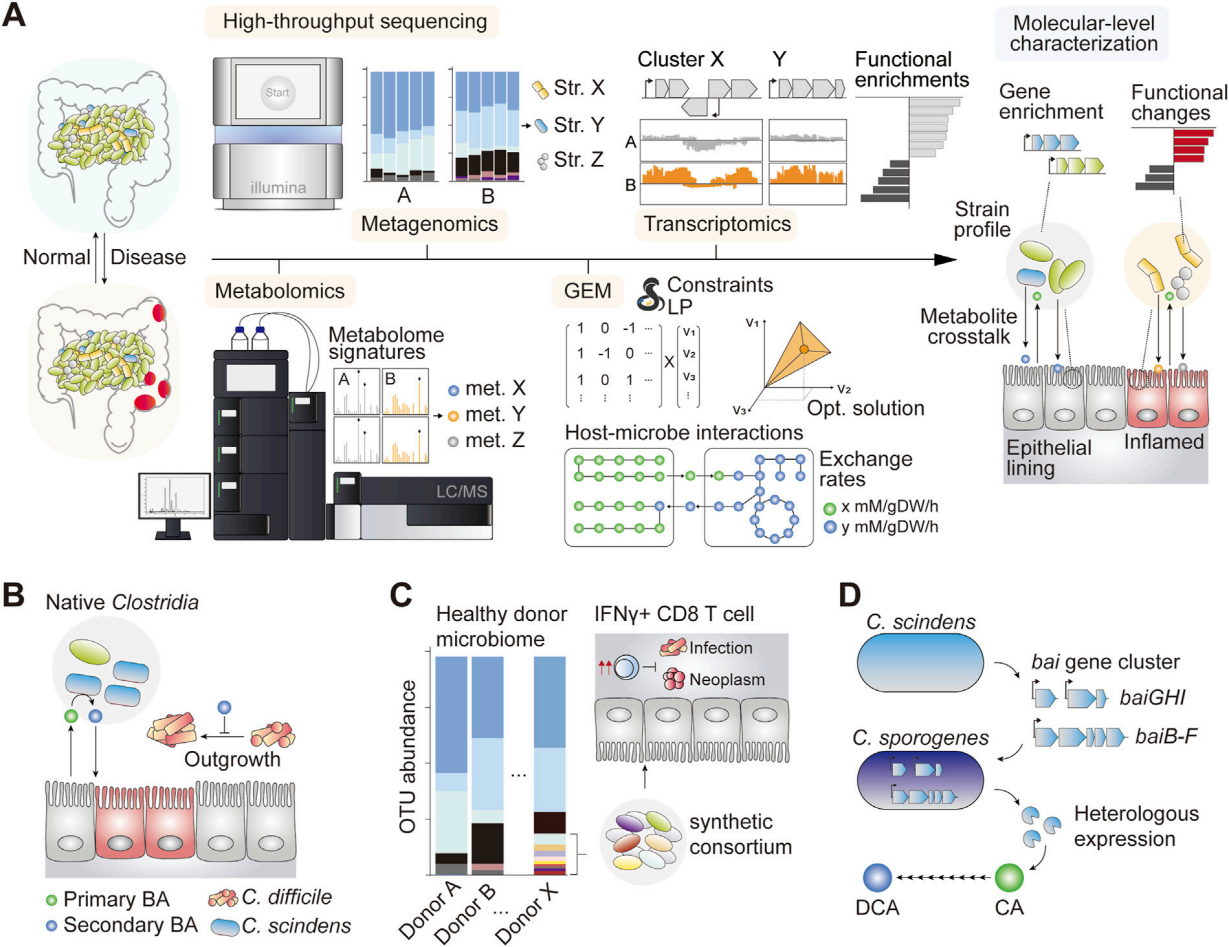
**(A)** Schematic of some notable systems biology approaches to understand the gut ecology and host–microbe interactions in the normal and inflamed gut **(B)** Introduction of *Clostridium scindens* and other causative species of *Clostridium* conferred *C. difficile* resistance phenotype primarily through the bioconversion of the luminal primary bile acid (BA) into the secondary form, which is inhibitory against *C. difficile* (Buffie et al., 2015) **(C)** A synthetically assembled consortium of 11 gut commensal bacteria induced proliferation of IFNγ CD8 T cells which showed anti-infective and anti-neoplastic effects in pre-clinical mouse models (Tanoue et al., 2019) **(D)** The *bai* gene cluster encoding primary BA deconjugation enzymes in *C. scindens* is heterologously expressed in genetically tractable species *C. sporogenes*, which functionally converts cholic acid (CA) into deoxycholic acid (DCA) *in vitro* (Funabashi et al., 2020).

The decade-long research on host-microbe interactions is now being translated into therapeutic product development. This is evident from the increasing number of microbe-based therapeutic regimens enrolling in clinical trials each year (Dronkers et al., 2020). In the next section, we provide a brief overview of the systems biology-driven approach to understanding the gut microbiota and outline how omics biology-driven analysis facilitates the exploration of host–microbe interactions at the molecular level. We also outline the ways in which the host-microbe interface is harnessed for potential therapeutic applications (Figure 1A).

### 2.1 Applications of systems biology in microbiome therapeutics

In April 2023, an investigational gut microbiota therapeutic tailored to address CDI, SER-109 (trade name VOWST), was approved by the Food and Drug Administration for clinical use (Mullard, 2023). This was the second FDA-approved microbiome therapeutic followed by a rectally administered microbiota cocktail, RBX2660 (trade name Rebyota). These two biotherapeutics have been tailored specifically for the prevention of CDI, which is a leading cause of antibiotic-associated diarrhea that results in 15,000–30,000 deaths annually (McDonald et al., 2018). Although the exact microbial compositions isolated from the therapeutic cocktails were not fully defined or disclosed (except that VOWST comprises selectively purified Firmicutes spores (Sims et al., 2023)), systems analysis of *C. difficile* etiology provides a glimpse into candidate species with potential therapeutic relevance and the underlying mechanisms of gut microbe-based modulation of the infection.

Gut microbiota dysbiosis is correlated with the depletion of luminal secondary bile acids (BAs) such as deoxycholic and lithocholic acids, which render the intestine highly prone to CDI (Theriot et al., 2016). The human gut microbiome, particularly the phyla Firmicutes and Bacteroidetes, have been predicted to harbor the biosynthetic capacity to transform host-derived primary BAs into 12 of the 13 types of secondary BAs *in vivo* (Heinken et al., 2019)*.* With regard to infection susceptibility, primary bile acids promote spore germination in *C. difficile,* whereas secondary BAs are inhibitory towards its vegetative growth and the TcdB toxin (Icho et al., 2023). Thus, the depletion of BA-converting species and other antipeptide-producing native gut flora during an antibiotic sweep creates an environment highly conducive to spore germination, overgrowth, and infection. In addition, secondary BAs activate orthogonal receptors that antagonize pro-inflammatory nuclear factor-κB (NF-κB) signaling (Collins et al., 2023). Therefore, the reduction in luminal secondary BA levels is thought to exacerbate gut inflammation and increase susceptibility towards spore-forming pathogenic agents, and antibiotic treatment frequently leads to relapses.

Systems biology has shed light on the therapeutic potential of gut microbiota editing for thwarting recurring CDI. In one study, authors identified native species of gut microbiota that contributed significantly to CDI resistance (Buffie et al., 2015). Importantly, species-level characterization of microbial abundance together with time-course modeling of microbial dynamics in response to antibiotic perturbation identified a group of bacteria that ameliorated infection upon engraftment (Figure 1B). Among these, the adoptive transfer of *Clostridium scindens*, a species that retains a complete secondary BA biosynthetic pathway (Funabashi et al., 2020), confers infection resistance (Buffie et al., 2015). Interestingly, subsequent analysis revealed that resistance to the *C. difficile* gene family was strongly linked to the enrichment of genes associated with secondary BA biosynthesis. The results were reproducible in a constraint-based microbial community modeling framework, where the simulation suggested that patients with inflammatory bowel disease (IBD) varied considerably in their metabolic capacity to produce secondary BAs compared with healthy controls (Heinken et al., 2019). Taken together, the systems analysis of *C. difficile* pathobiology helped identify a potent probiotic candidate as well as the underlying mechanism of action, ultimately allowing novel therapeutic options for difficult-to-treat diseases such as CDI (with the FDA-approved therapeutics Rebyota and VOWST).

Recent progress in etiological characterization of celiac disease (CD) driven by-multi-omics analysis is also worth notable mentions (Leonard et al., 2020; Leonard et al., 2021). CD is an autoimmune condition triggered by gluten consumption. While it has been proposed that genetic predisposition and environmental stimuli play key role in CD development, its exact pathogenesis and etiology remains incompletely understood. Recently the Celiac Disease Genomic, Environmental, Microbiome, and Metabolomic (CDGEMM) study was launched in an attempt to investigate potential link between gut microbiota and CD development. The metagenomic and metabolomic features in stools collected from a longitudinal cohort of infants at high risks of CD were analyzed to explore multivariate risk factors encompassing genetics, mode of delivery and feeding, antibiotic use to CD development, in the venture point of gut microbiome (Leonard et al., 2020). Interestingly, the study reported major taxonomic and functional shifts in the gut microbiota of CD-prone infants, providing insights on how environmental factors that shape gut microbiota composition, in turn, may adversely affect immunomodulation of the host (Leonard et al., 2020). More recently, a longitudinal study that enrolled 10 CD and 10 non-CD infants reported changes in the gut metagenomes, pathway enrichments and associated metabolomes that are specific to the onset of CD (Leonard et al., 2021). As such, the multi-omics driven investigation of the longitudinal changes in gut microbiome has yielded valuable insights into the emerging role of gut microbiota in the development the chronic autoimmune disease. The data-driven findings shed light onto potentially novel therapeutic targets for the treatment and prevention of CD. The efforts are currently ongoing, with more than 550 subjects enrolled in the study as of May 2023. The large-scale longitudinal investigations hold the promise of unveiling undercharacterized molecular mechanisms and bring forth innovative therapeutic modalities for addressing CD (Leonard et al., 2023).

Synthetic microbial consortia designed with careful consideration of strain-specific metabolism, potential impact on host physiology, and safety profiles have been demonstrated to be a viable and effective therapeutic modality for different disease types (Table 1). In a study investigating CDI treatment, researchers developed a formula of synthetic microbial consortia with a defined species composition, using microbial isolates obtained from a healthy donor stool. This synthetic regimen was effective in preventing recurrent CDI in affected patients for up to 6 months (Petrof et al., 2013). In another study, a group of bacterial species that stimulated the proliferation of interferon-γ-secretory CD8 T cells demonstrated a potent therapeutic effect against infectious and neoplastic diseases in murine models (Figure 1C) (Tanoue et al., 2019). In this study, synthetic consortia comprising low-abundance species of healthy human microbiota exhibited enhanced clearance of the infectious agent *Listeria monocytogenes* and potent anti-tumor effects without observable adverse effects (Tanoue et al., 2019). The study underscored the importance of underrepresented species in omics data, which nonetheless have broad implications in host physiology, emphasizing the need to revisit sequence-oriented microbiome analysis weighted heavily on the quantitative evaluation of individual components.

**TABLE 1:** Notable examples of native or synthetic microbe consortia in therapy.

Composition	Target disease/physiology	Mechanism of action	Model system	Ref
Selectively purified firmicutes spores from healthy human donors	CDI	Exact mechanism undefined. Possibly involves competitive exclusion of *C. difficile* presumably through secondary BA production (given the presence of firmicutes phyla)	Approved for human use (trade name VOWST)	Sims et al. (2023)
Microbiota suspension from healthy human donors	CDI	Competitive exclusion of *C. difficile* through an undefined mechanism	Approved for human use (trade name Rebyota)	Adolfsen et al. (2021)
Defined consortium of 33 gut commensals	CDI	Competitive exclusion of *C. difficile* through an unknown mechanism	Human clinical trial (NCT01372943)	Petrof et al. (2013)
Defined consortium of 11 gut commensals	Tumor, infection (immunomodulation)	Bacterial colonization, and bacterial antigen-mediated induction of interferon-γ-secretory CD8 T cells in colonic epithelial cells	Mouse	Tanoue et al. (2019)
*C. scindens* and engineered *Bacteroides* species (*B. thetaiotaomicron*, *B. fragilis*, or *B. ovatus*)	Immunomodulation	Induction of CD4^+^ regulatory T cells Treg cells	Mouse	Campbell et al. (2020)
		Two step synthesis of 3β-hydroxydeoxycholic acid production from cholic acid via (i) cleavage of the 7α-hydroxyl group in cholic acid by *Clostridium scindens*, followed by (ii) epimerization of 3α-hydroxyl group in deoxycholic acid by engineered *Bacteroides* expressing hydroxysteroid dehydrogenases of *Ruminococcus gnavus* origin		

It is also worth noting that the application of systems biology extends to functional genetics, enabling the characterization of the aforementioned causal factors at nucleotide-level. For example, functional coding sequences of the complete secondary BA biosynthetic pathway of intractable gut microbes have been published recently (Funabashi et al., 2020; Sato et al., 2021), paving the way for functional expression and control of secondary BA biosynthetic pools for therapeutic applications (Figure 1D) (Funabashi et al., 2020; Koh et al., 2022). While the multitudes of systems biology toolkits have drastically expanded our ability to characterize host-microbe interactions in unprecedented detail, our ability to effectively manipulate and engineer gut commensals remains a challenge. Genetic manipulation of gut commensal species is important for validating data-driven hypotheses and serves as an enabling technology in the growing field of live microbial therapeutics. In this section, we describe a novel therapeutic modality driven by synthetic biology.

## 3 Engineering microbes for therapeutic applications

Repurposing individual microbes for therapeutic applications, referred to as live bacterial therapeutics (LBTs), requires a robust chassis strain with the ability to survive, thrive, and drive the production and delivery of the desired payloads *in vivo* (Inda et al., 2019). Furthermore, the need for Generally Recognized as Safe (GRAS) makes probiotic bacteria that are harmless, beneficial to human health, capable of thriving in the host, best suited for therapeutic applications. The earliest and most prominent example was the use of the probiotic strain *Escherichia coli* Nissle 1917 (EcN), which has clinically relevant antimicrobial and immunomodulatory properties (Schultz, 2008; Sassone-Corsi et al., 2016). Since then, the administration of various probiotics with beneficial properties has been the treatment of choice for gut inflammation and dysbiosis (Fukuda et al., 2011; Sassone-Corsi et al., 2016).

Despite the reported efficacy and valid conceptual basis for its therapeutic effects (Schultz, 2008), probiotic treatment often leads to conflicting prognoses and is not recommended for therapeutic use by some medical organizations (Dickson, 2019; Guandalini and Sansotta, 2019). This calls for genetic engineering interventions to augment the beneficial properties of probiotics and confer novel clinically relevant phenotypes. The expanding availability of novel bioparts and genetic toolkits has allowed the programming of a microbial therapeutic chassis for IBD, cancer therapy, and synthetic metabolism (Lai et al., 2022; Lynch et al., 2022). Although the efficacy of most biotherapeutic projects has been limited to laboratory settings, increasing numbers of biotherapeutics are being evaluated for clinical applications (Dronkers et al., 2020). In this section, we describe the conceptual basis of LBT engineering, latest updates, and emerging use of gut commensal bacteria as a next-generation biotherapeutic chassis.

### 3.1 Synthetic biology approach to LBT construction

**FIGURE 2:**
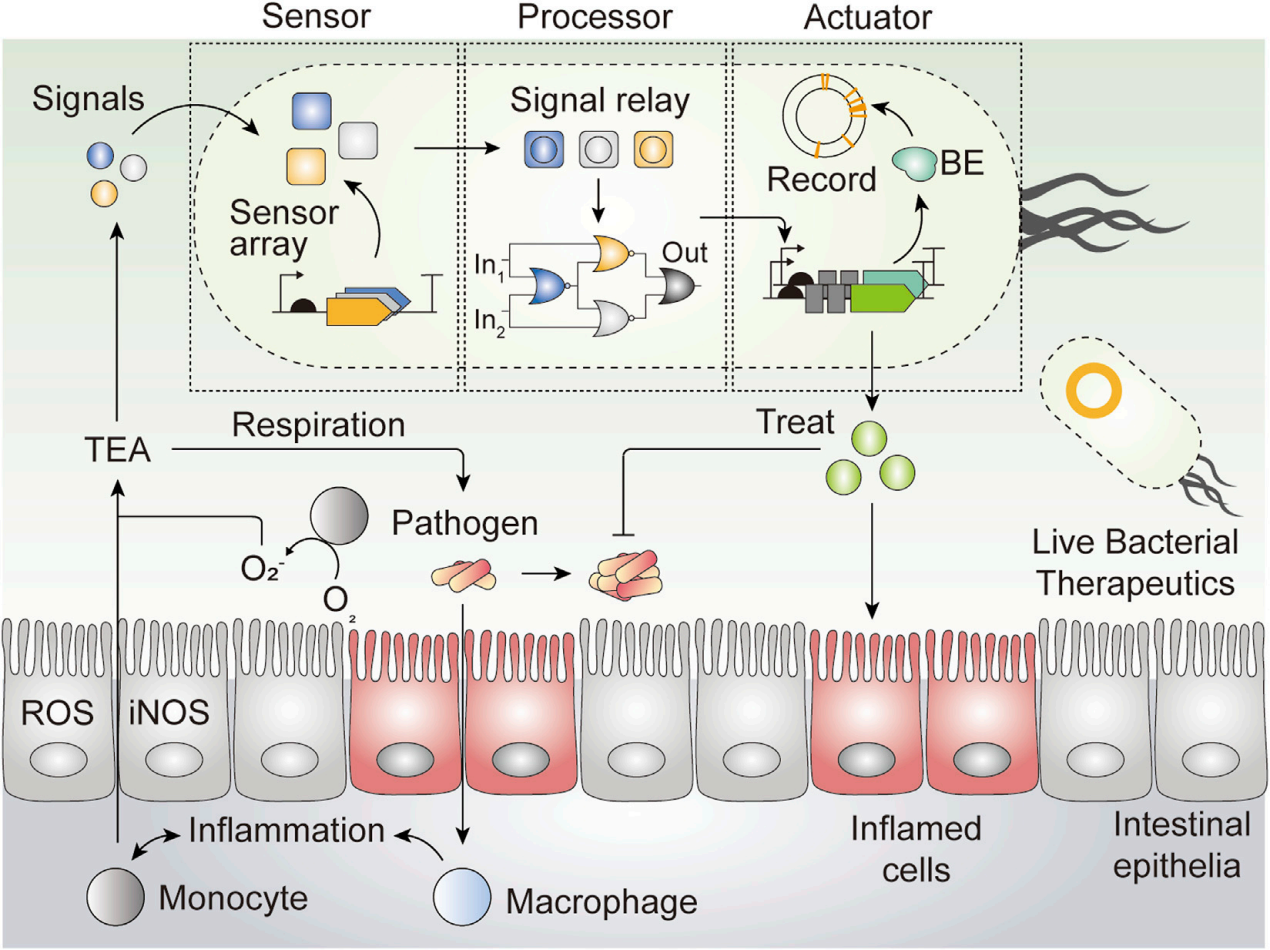
Schematic of engineered live bacterial therapeutics. An LBT can simultaneously sense surrounding input signals, and process the signals using elaborate Boolean logic gates, which are relayed to guide response outpuslt. By tethering the output signals with appropriate actuator genes, an LBT can execute user-programmed functionalities such as genetic memory (with base editor; BE), and therapeutic delivery*, in vivo*. Abbreviations–TEA: terminal electron acceptor, BE: base editor.

Assured safety, advanced genetic tractability, and relative ease of manipulation have led to the expanding use of probiotics as LBTs, which are live microbes engineered to deliver therapeutic payloads *in vivo* (Steidler et al., 2000). The development of titratable substrate-specific promoter arrays (Meyer et al., 2019) that facilitate complex logic operations (Nielsen et al., 2016; Jones et al., 2022) has streamlined the design and engineering of LBTs that perform real-time detection and therapeutic payload delivery in response to specific signals *in vivo* (Triassi et al., 2023). An LBT chassis can simultaneously diagnose, record, and self-regulate therapeutic payload delivery in response to host disease *in vivo* (Figure 2) (Zou et al., 2023). Such ‘smart LBT,’ for lack of a better term, is powered by versatile genetic circuitry that constitutes independent genetic components, each of which enables sensing of environmental cues, signal relay and processing, and actuation (Figure 2). Each genetic component is modularized as composite bioparts to enable reconfiguration of cellular functions in a “plug-and-play” manner; that is, as far as chassis strain is concerned, cells can be reprogrammed to execute various user-defined operations by re-wiring different input signals to output modules.

Biosensor modules, such as endogenous transcription factors (TFs), enzymes, or nucleic acids, regulate gene expression. The mode of action of a genetic biosensor typically entails a conformational change upon ligand binding, followed by the activation of orthogonal transcription machinery. Using a two-component signal transduction system (TCS) as an example, the membrane-bound sensor histidine kinase ThsS phosphorelays thiosulfate (S_2_O_3_^2−^)-induced signals at the receiving end of the DNA-binding regulator ThsR. Phosphorylated ThsR, in turn, activates its cognate promoter P_phsA_ and expression of the downstream reporter gene superfolder GFP (sfGFP) to transform the ligand-binding signal into luminescence readouts in a dose-dependent manner (Daeffler et al., 2017). To date, an array of genetic biosensors specific to the host and/or disease-specific biomarkers has been reported. These include biosensors for inflammation, infection, bleeding, cancer, diabetes, and availability of abiotic factors such as pH and O_2_ (Table 2). The sensing modules confer stringent control over LBT phenotypes and augment the performance. In one instance, a refactoring of K5-type capsular polysaccharide expression with the *lac* promoter in EcN allowed programmable surface antigen display using isopropyl-b-D-thiogalactopyranoside (IPTG) induction. This rather simple tweak in transcription regulation led to prolonged *in vivo* survival by allowing programmable evasion of the host immune response, consequently augmenting the antitumor efficacy of LBT by ten-fold (Harimoto et al., 2022). The integration of more than one transcriptional regulator in a complex cellular response is characterized by multimodal transgene expression in response to diverse environmental cues (Taketani et al., 2020).

**TABLE 2:** Examples of LBTs for disease diagnosis and/or treatment.

Condition	Biomarker	Genetic sensor/inducer system(s)	Actuator(s)/other synthetic feature(s)	Chassis	Model systems	Ref
IBD	Thiosulfate	ThsSR-P_*phsA*_ TCS-cognate promoter pair for thiosulfate detection and signal relay	sfGFP, hly-avCys (signal peptide-linked AvCystatin) reporter, therapeutic payload modules BE2-sgRNA based editor module	EcN	Mouse	Zou et al. (2023)
	Thiosulfate, tetrathionate	ThsSR-P_*phsA*_ and TtrSR-P_*ttrB*_ to sense thiosulfate and tetrathionate, respectively	sfGFP reporter	EcN	Mouse	Daeffler et al. (2017)
	Nitrate, thiosulfate	NarXL-P_yeaR_ to sense nitrate. Boolean AND logic gate by using additional ThsSR-P_*phsA*_ and the nitrate sensor module	sfGFP reporter Δ*narXL* (deletion of native NarXL)	EcN	Mouse	Woo et al. (2020)
	Tetrathionate	Tetrathionate sensor TtrSR with cognate promoters P_*ttrSR*_ and P_*ttrBCA*_	Cro-inducible CI/Cro bistable switch-based cell memory record	*E. coli*	Mouse	Riglar et al. (2017)
				NGF-1		
	Nitric oxide	Enhancer binding protein NorR and the cognate promoter P_*norV*_	DNA recombinase *fimE* to control bidirectional CFP (cyan fluorescence protein), YFP (yellow fluorescence protein) reporter switch	*E. coli* TOP10	Mouse ileum explant culture	Archer et al. (2012)
	Calprotectin	Zinc-responsive native promoter of *ykgMO*	sfGFP reporter	EcN	Mouse	Xia et al. (2023)
	Dietary xylan	*B. ovatus* xylanase promoter (xylanase operon)	murine interleukin-2 (MuIL2)	*B*. *ovatus*	*In vitro* culture	Farrar et al. (2005)
		*B. ovatus* xylanase promoter (GenBank EU334491)	Human keratinocyte growth factor-2 (hKGF-2)	*B*. *ovatus*	Mouse	Hamady et al. (2010)
		Identical to Hamady et al. (2010)	Human transforming growth factor-β (hTGF-β)	*B*. *ovatus*	Mouse	Hamady et al. (2011)
CDI	Sialic acid	Sialic acid sensor NanR-P_*NanA*_ and a transcriptional activator CadC*-*P_*CadBA*_ (signal amplifier)	Cbh (Choloyglycine hydrolase) under the control of P_*CadBA*_	EcN	Mouse	Koh et al. (2022)
*V. cholerae*	Cholerae autoinducer-1 (CAI-1)	A chimeric sensor composed of the membrane binding domain of CqsS (the CAI-1 receptor) and the signal transduction domain of NisK (the signal transduction domain of NisRK TCS), with cognate P_*nisA*_	TetR-inducible transcriptional inverter circuit containing either mCherry or β-lactamase gene	*Lactococcus lactis*	Mouse	Mao et al. (2018)
		A phosphorelay system of CqsS, LuxU and LuxO that controls the activity of tpqrr4 promoter	guide RNA (gRNA) for the repression of ‘lysis and kill’ pBAD-YebF-Art-085 cassette, upon binding with constitutively expressed dCas9	*E. coli* MG1655	*In vitro* culture	Jayaraman et al. (2017)
*P. aeruginosa*	N-acyl homoserine lactone (AHL)	P_*tetR*_ based expression of AHL-sensing transcription factor LasR, which activates its cognate promoter P_*luxR*_	Pyocin S5, and Lysis E7	*E. coli* TOP10	Co-culture *in vitro*	Saeidi et al. (2011)
		The ‘sense-kill’ genetic system identical to Saeidi et al. (2011)	Pyocin S5, Lysis E7, and Dispersin B. D-ala auxotrophic genotype Δ*alr* Δ*dadX* for plasmid maintenance	EcN	*C. elegans*, mouse	Hwang et al. (2017)
Methicillin-resistant *Staphylococcus aureus* (MRSA)	Autoinducer peptide-I (AIP-I)	AIP-I sensing TCS AgrCA and the cognate P3 promoter	GusA reporter	*Lactobacillus reuteri*	*In vitro* culture	Lubkowicz et al. (2018)
Enterococcal infection	Enterococcal sex pheromone cCF10	cCF10-responsive regulator PrgX that activates promoter P_Q_ upon cCF10 binding	Anti-enterococcal peptides EntA, EntP, HirJM79	*L. lactis*	*In vitro* culture	Borrero et al. (2015)
*Citrobacter rodentium*	Fucose	Promoter region upstream of *E. coli fucPIK* genes	GFP reporter	*E. coli* BW25113	Mouse	Pickard et al. (2014)
Gastrointestinal health	Heme, AHL, thiosulfate	Heme-responsive transcriptional repressor HrtR and the cognate synthetic promoter P_*L(HrtO)*_, or AHL-sensor LasR- P_*luxR*_, or thiosulfate sensor ThsSR-P_*phsA*_	LuxCDABE luciferase and a wireless luminometer that relays luminescence into electric readouts (ingestible micro-bio-electronic device; IMBED). ChuA for extracellular heme transit	EcN	Mouse, swine	Mimee et al. (2018)
Liver metastasis	IPTG	IPTG-inducible expression system LacI-P_*tac*_	LacZ reporter	EcN	Mouse	Danino et al. (2015)
Tumor	Tumor-specific agent(s)	Tumor-responsive regulatory elements in *Salmonella* Typhimurium genome screened using the promoter-trap library	GFP_ovalbumin low-stability reporter	Attenuated *Salmonella enterica* subsp. enterica *serovar* Typhimurium	Mouse, colon carcinoma cells	Leschner et al. (2012)
	IPTG	IPTG-inducible expression system LacI-P_*tac*_	Episomally-maintained IPTG-inducible KfiC in Δ*kfiC* genomic background	EcN	Mouse	Harimoto et al. (2022)
	Anoxic condition	Anaerobic-inducible FNR-P_*fnrS*_	DacA (STING-agonist cyclic di-AMP), Δ*thyA*, Δ*dapA*	EcN	Mouse, human antigent-presenting cells	Leventhal et al. (2020)
	Lactic acid, anoxic condition, pH	Lactic-acid inducible LldR-P_*LldR*_, anaerobic-inducible FNR-P_*fnrS*_, pH-sensitive CadC-P_*CadC*_ expression systems	GFP reporter, Δ*asd*, Δ*glms*	EcN	Tumor condition-mimicking spheroid coculture system, mouse	Chien et al. (2022)
		Lactate-hypoxia Boolean AND logic gate to enhance tumor-specific localization				
	AHL	A synchronized lysis circuit using coupled positive and negative feedback loops, using P_*luxl*_ that controls LuxI expression	Positive feedback: LuxI under the control of P_*luxl*_ produces AHL, which binds constitutively produced LuxR to drive P_*luxl*_ expression	Attenuated *S*. Typhimurium	Mouse	Din et al. (2016)
Inflammation, diabetes	Nitrogen oxides, glucose	Nitrate/nitrite-sensitive transcriptional promoter P_*YeaR*_, glucose sensitive promoter P_*CpxP*_	Negative feedback: cell death triggered by bacteriophage lysis gene (φX174 E). sfGFP reporter and HlyE (hemolysin)	BxB1 and TP901 integrase (genetic switches)-mediated Boolean logic gates, GFP and RFP as actuators	*E. coli* DH5αZ1 Urine, serum samples	Courbet et al. (2015)
*Bacillus subtilis* Hyperammonemia	Anoxic condition	Anaerobic-inducible FNR-P_*fnrS*_	ArgA^fbr^., Δ*thyA*, Δ*argR*	EcN	Mouse	Kurtz et al. (2019)
Enteric hyperoxaluria	Anoxic condition	Anaerobic-inducible FNR-P_*fnrS*_	OxlT, OxdC, Frc, ScAAE3, Δ*thyA*	EcN	Mouse	Lubkowicz et al. (2022)
Homocystinuria	IPTG	IPTG-inducible expression system LacI-P_*tac*_	MetD, MetC, MetP, Δ*yjeH*	EcN	Human clinical trial (NCT05462132)	Perreault et al. (2021)
PKU	IPTG, L-arabinose, anoxic condition	IPTG-inducible LacI-P_*tac*_, L-arabinose inducible AraC-P_*BAD*_, anaerobic-inducible FNR-P_*fnrS*_	PAL (5 copies), PheP (2 copies), LAAD, Δ*dapA*	EcN	Human clinical trial (NCT04534842)	Isabella et al. (2018)
		Identical to Isabella et al. (2018)	Identical to Isabella et al. (2018) except for a mutant PAL (mPAL)	EcN	Human clinical trial (NCT05764239)	Adolfsen et al. (2021)
		PhlF-P_*PhlF*_ repressor, IPTG-inducible LacI-P_*tac*_, AraC-P_*BAD*_	mPAL (3 copies) and PheP (1 copy) under P_*PhlF*_ control. LAAD under P_*BAD*_ control, Δ*dapA*	EcN	*In vitro* culture	Triassi et al. (2023)

Complex cell programming is achieved through signal processing modules, which are important for informing LBTs about specific locations and timings to produce therapeutic responses at the required dosage. To this end, Boolean logic gates that compute input signals to guide appropriate responses have been implemented in genetic circuit designs. For instance, the biomarkers of gut inflammation thiosulfate/tetrathionate (S_4_O_6_^2−^) and nitrate (NO^3−^) are proxies for two distinct etiologies of IBD (Winter et al., 2010; Winter et al., 2013), and the means to detect each biomarker alone may be insufficient for IBD diagnosis. To address this issue, a genetic circuit incorporating an AND Boolean logic gate was constructed based on two TCSs and cognate promoter pairs, ThsSR with P_*phsA*_, and NarXL with P_*yeaR*_; creating a system that requires the presence of two IBD biomarkers, thiosulfate and nitrate, to elicit an actuator response (Woo et al., 2020). The use of complex logic operations further enhances the specificity of genetic circuits by imparting multilevel control to the system output. However, designing genetic circuits with an increasing number of control elements is difficult and time consuming, which limits the implementation of complex cell programming (Kwok, 2010). Automated genetic circuit design software (Cello) streamlines the entire workflow (Nielsen et al., 2016; Jones et al., 2022). The software allowed the functional design of a genetic circuit using a library of NOR/NOT gates as complex as 10 regulators and 55 parts. In addition, the response functions of the sensors and gates can be extrapolated to quantitatively predict the behavior of the designed genetic sensor. A reliable circuit design algorithm that boasts 92% circuit output prediction accuracy was applied in the design of a gut commensal chassis for multimodal transgene expression (Taketani et al., 2020) as well as in an LBT to enable timely, predictable expression of therapeutic payloads in a condition-specific manner (Triassi et al., 2023).

### 3.2 Enhancing LBT performance for clinical applications

Fine-tuning of transgene expression and the activity of encoded products are commonly sought to optimize strain performance. It should be noted that the functional expression of transgenes per the intended design may require extensive troubleshooting and *ad hoc* optimizations. For instance, owing to the highly subjective nature of composite elements within a genetic circuit, determining the optimal parameters for an expression system is confounded by multiple variables, such as the strength of the expression machinery (Chien et al., 2022; Choe et al., 2022; Song et al., 2022) and genetic and environmental contexts (Lou et al., 2012). In many instances, an optimal system output can be achieved by shuffling genetic parts (Choe et al., 2022) or their brute-force combinations (Song et al., 2022) and copy number variations (Isabella et al., 2018) to fine-tune the target transgene expression. Enhancing the activity of the encoded functions is also an integral part of the optimization process, particularly when transgene expression is not a bottleneck. To this end, the evolutionary (directed) engineering of target proteins at a specific scale, followed by biosensor-assisted screening, is effective in identifying target proteins with improved functionalities (Adolfsen et al., 2021). Furthermore, the characterization of highly expressed genomic regions within the chassis strains can be repurposed as a ‘landing pad’ for transgene expression cassettes, which confers higher genetic stability over the plasmid-based expression regimes (Park et al., 2020).

**FIGURE 3:**
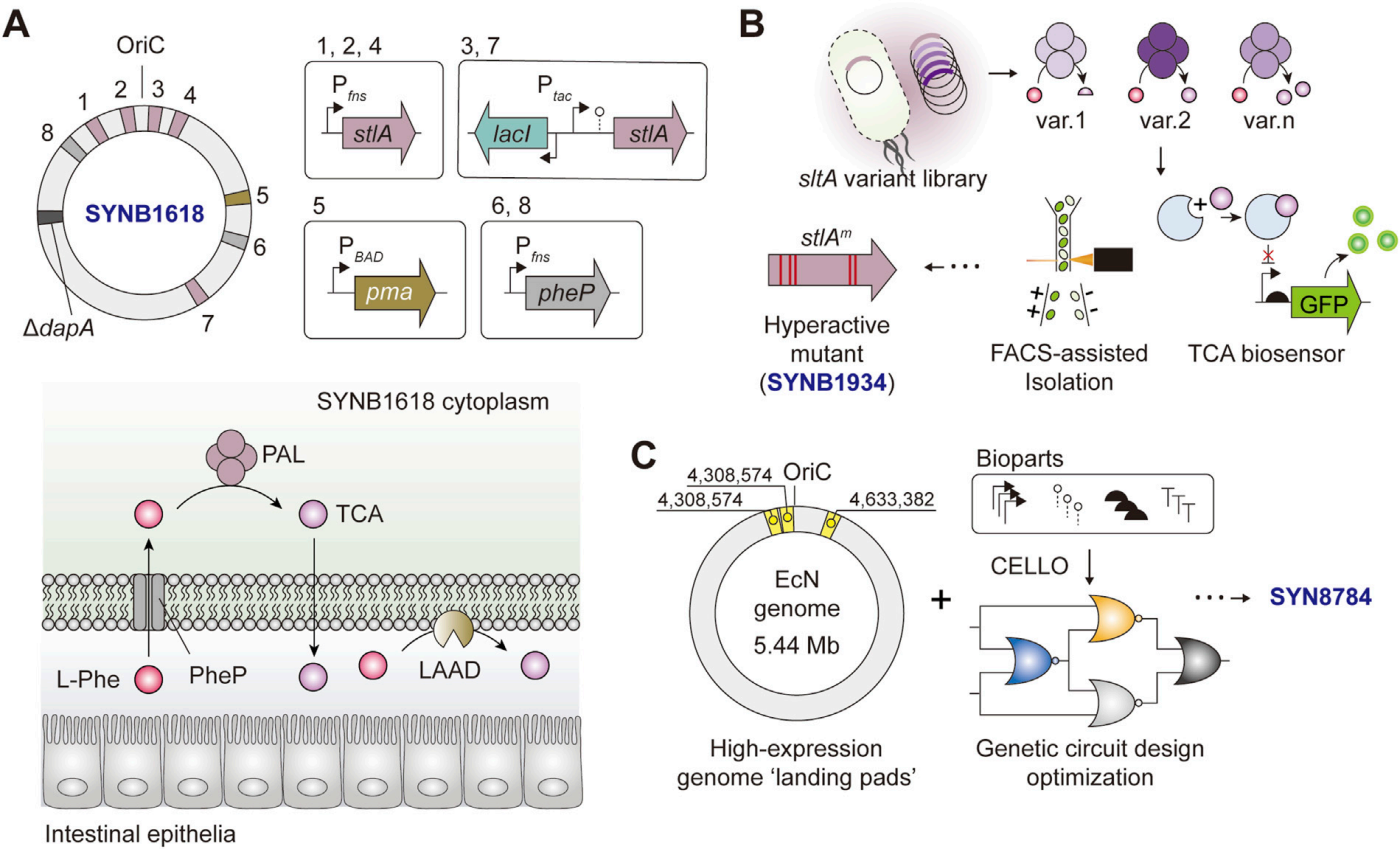
Simplified schematics of *Escherichia coli* Nissle 1917-based LBTs tailored for the treatment of PKU **(A)** SYNB1617 harbors five copies of *stlA*, two copies o f *pheP*, and a single copy of *pma* under the expression of P_*tac*_, P_*BAD*_, or P_*fnrS*_. The deletion of 4-hydroxy-tetrahydrodipicolinate synthase (*dapA*) renders the bacteria auxotrophic to diaminopimelate (DAP) **(B)** A simplified workflow for screening and isolation of *sltA* ^*m*^ encoding “hyperactive” PAL mutants, the key component of SYNB1934 **(C)** Engineering logic behind SYN8784 construction.

SYNB1618 and its derivatives are LBTs designed for the treatment of phenylketonuria (PKU) and assimilate the aforementioned engineering and optimization principles, which are worthy of a detailed discussion (Figure 3). The original strain, SYNB1618, is an EcN-based LBT engineered to complement a disabling mutation in the host phenylalanine hydroxylase (PAH), which is the cause of the debilitating metabolic disease. SYNB1618 carries two synthetic phenylalanine degradation pathways. First, PheP and PAL, encoded by phenylalanine transporter, *pheP* and phenylalanine ammonia-lyase, *stlA*, respectively. Second, LAAD, encoded by membrane-associated L-amino acid deaminase *pma* (Figure 3A) (Isabella et al., 2018). To achieve high expression levels and impart transcriptional control *in vivo*, multiple copies of *pheP*/*stlA* were genome-integrated and expression-controlled in an IPTG- (P_*tac*_), L-arabinose (P_*BAD*_) or anaerobic-inducible (P_*fnrS*_) manner (Figure 3A). The *in vivo* analysis showed that SYNB1618 was effective in lowering blood phenylalanine levels in a mouse model of PKU, stabilized phenylalanine levels in healthy non-human primates (Isabella et al., 2018), and was safely tolerated by healthy individuals (Puurunen et al., 2021).

In a follow-up study aimed at further augmenting the phenylalanine-degrading capacity of LBT, PAL was no longer rate-limiting, hence calling for efforts to optimize the performance of PAL (Adolfsen et al., 2021). In this study, the authors used a biosensor-assisted screen based on an allosteric TF activated in the presence of *trans*-cinnamate (TCA), a product of PAL, in a concentration-dependent manner. The *stlA* mutant library was designed considering the enzyme phylogeny, co-evolution, and structural attributes to infer enzyme residues for targeted engineering. To avoid cross-talk between different TCA producer strains (which also house the TF-based biosensor) in the pooled library, an oil-emulsion-assisted microculture followed by a fluorescence-activated cell sorting (FACS) strategy was adopted to effectively isolate high TCA-producing mutants (Figure 3B) (Adolfsen et al., 2021). The authors identified a PAL mutant (stlA* or mPAL) carrying five mutations (S92G, H133M, I167K, L432I, and V470A) with *V*_max_ and *K*_*M*_ two to three folds higher compared to that of wild-type PAL. The SYNB1618-derivative that expresses stlA*, designated SYNB 1934, showed an approximately 2-fold increase in *in vivo* phenylalanine conversion capacity compared to SYNB1618 (Adolfsen et al., 2021). The PKU-targeting LBT series SYNB1618 and SYNB1934 have undergone phase I and II clinical trials (ClinicalTrials.gov ID: NCT04984525 and NCT04534842, respectively), and a phase III trial of SYNB 1934 (NCT05764239) is underway in June 2023.

More recently, an independent study revisited the transgene expression architecture of SYNB1618 with the final goal of optimizing its therapeutic gene expression and alleviating the potential genetic burdens incurred during the process. By leveraging the knowledge of genomic ‘hot spots’ that are conducive to strong gene expression and fine-tuning transgene expression using automated genetic circuit design, the authors constructed the final strain, SYN8784 (Figure 3C). With fewer genomic copies of stlA* and *PheP*, SYN8784 maintained higher TCA activity while also exhibiting enhanced strain fitness compared to SYNB1618, underscoring the importance of rationalized genetic circuit design informed by computer-aided design tools and augmentation of strain functions to reinforce system performance (Park et al., 2020; Triassi et al., 2023).

Together, these studies show that a synthetic-biology-powered chassis platform can be readily translated into real-life applications. The use of modularized bioparts with quantitatively measured and standardized characteristics confers enormous flexibility and predictability to the biological function design, facilitating the reconfiguration of cellular phenotypes in a user-defined manner. However, our insufficient understanding of complex biology limits our capacity to overcome certain biological constraints. The inherent biological constraints of probiotic strains, such as rapid clearance, low *in vivo* cell density, and inability to colonize the intestinal niche for an extended duration, represent hurdles in probiotic-based therapeutics (Zmora et al., 2018), especially for addressing chronic diseases (Inda et al., 2019). As a promising alternative, repurposing native gut microbes for therapeutic applications has gained traction in recent years. In the next section, we discuss systems and synthetic biology approaches for repurposing native gut resident species for therapeutic purposes.

## 4 Engineering gut commensal species for therapeutic applications

Repurposing the gut microbiota for various applications follows the same engineering principles. However, such attempts have been hampered by the incompatibility of existing molecular toolkits and the relative scarcity of relevant resources to manipulate genomes in the past (Salyers et al., 2000). In recent years, a suite of multi-omics and bioinformatics analytic pipelines has led to the discovery of unique transcription architectures as well as regulatory components that can be repurposed as transgene expression tools. In addition, continued efforts to harness the gut microbiota will expand the genetic toolbox for engineering individual commensal species.

In particular, *Bacteroides* species represent some of the most genetically amenable and extensively engineered gut commensals. Recent reviews/proceedings on microbiome (in particular, *Bacteroides*) engineering highlights the “technological readiness level” of engineering gut commensals that is nearly on par with that of other model species (Lai et al., 2022; McClure et al., 2022). For example, the availability of validated bioparts enables functional design and implementation of artificial genetic circuits to elicit multimodal actuator responses in individual gut commensals and their consortia, demonstrating programmed sense-and-respond operations *in vitro* and *in vivo* (Taketani et al., 2020; Huang et al., 2022). Remarkably, an artificially assembled porphyran PUL gene segment measuring 60 kb was transplanted into the *Bacteroides thetaiotaomicron* genome to enable the utilization of the polysaccharide porphyran, a carbohydrate found in seaweed that is inaccessible to the native gut microbiota (Shepherd et al., 2018). The colonization efficiency and *in vivo* abundance of engineered *B. thetaiotaomicron* can be controlled exogenously by porphyran supplementation, which is a promising development for stable engraftment and control of LBTs *in vivo* (Shepherd et al., 2018). Owing to this superior genetic tractability and numerical predominance of the species across individuals, *Bacteroides* in their native and engineered forms have been used in a host of gut microbiome studies, which ultimately led them to earn the status of, as Wexler and Goodman put it, “a window into the microbiome” (Wexler and Goodman, 2017).

**FIGURE 4:**
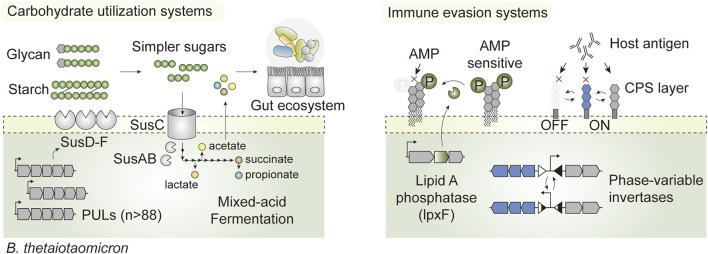
Hallmark features that promote *in vivo* survival in the prominent gut commensal *Bacteroides thetaiotaomicron*. The presence of exhaustive carbohydrate-active enzymes encoded by PULs enables foraging on complex dietary carbohydrates and host glycans (Starch utilization systems; SusA-F for illustration purposes). This not only confers a competitive edge for survival through the feast and famine cycles in the intestine, but it also shapes the luminal nutrient (or metabolic) landscape by converting otherwise inaccessible carbohydrates into simpler oligosaccharides and short chain fatty acids that can be utilized by the native gut flora and host epithelia. *B. thetaiotaomicron* also houses phase variable invertases and phosphatases that dynamically shift cell surface architectures in response to environmental perturbation, promoting evasion of the host immune system and exogenous antimicrobial peptides (AMP).

*Bacteroides* possess an ensemble of adaptive features that are advantageous for *in vivo* survival. First, it encodes a broad class of carbohydrate-active enzymes that allow foraging on different forms of carbohydrates, from complex dietary glycans to host-derived glycoproteins (Martens et al., 2008; Luis et al., 2018), which confer survival advantages through the feast and famine cycles. Specifically, *B. thetaiotaomicron* expresses an elaborate repertoire of saccharolytic enzymes via specific gene clusters known as polysaccharide utilization loci (PUL), which together account for nearly 18% of the whole genome (Xu et al., 2003). There are as many as 88 such loci, each embedded with at least one homolog of *susC* and *susD*, which enable *B. thetaiotaomicron* to utilize a broad range of substrates (Martens et al., 2008) (Figure 4). In addition, phase-variable systems controlling surface antigen expression enable the evasion of host immunity and phage infection (Cullen et al., 2015; Taketani et al., 2015; Porter et al., 2020), and promote phenotypic diversity that mediates colonization in the intestine (Jiang et al., 2019) (Figure 4). Such intrinsic features enable native members of the gut microbiota to stably colonize the gut ecosystem, occupy specific luminal niches such as glands and crypts, and propagate autonomously at high density for as long as a decade (Faith et al., 2013; Lee et al., 2013). Therefore, gut commensal-based LBTs may be better suited than their probiotic counterparts to address chronic diseases or those that require long-term interventions (Inda et al., 2019).

In addition to the adaptive features that favor gut colonization, mounting evidence highlights the therapeutic relevance of native *Bacteroides* and other commensals in human health and disease, including beneficial systemic effects on host metabolism and immunomodulation in inflammation, infection, and cancer therapy (Buffie et al., 2015; Plovier et al., 2017; Tanoue et al., 2019). Notably, *B. thetaiotaomicron* alleviated gut inflammation in a human Caco-2 infection model by selectively antagonizing pro-inflammatory NF-κB signaling through the nuclear export of the transcriptionally active NF-κB subunit RelA into the cytoplasm, thereby preventing downstream inflammatory cascades (Kelly et al., 2004). The protective effects of *B. thetaiotaomicron* extend to murine models of colitis, characterized by reduced weight loss, histopathological damage, and inflammation (Delday et al., 2019). Interestingly, treatment with purified pirin-like protein (BT0187) in the Caco-2 cell lines led to reduced NF-κB levels and reproduced several hallmarks of therapeutic effects in live cells, suggesting its potential therapeutic use for inflammatory diseases (Delday et al., 2019). Recently, an assessment of the safety and tolerability status of *B. thetaiotaomicron* suggested that the strain was well tolerated by patients with Crohn’s disease (NCT02704728) (Hansen et al., 2021), further underscoring the applicability of beneficial gut commensals in LBTs. In this section, we describe the systems and synthetic biology-guided design and engineering of LBTs using the prominent native gut commensal *Bacteroides* as a model. A host of other gut inhabitants and probiotics species are also being employed for LBT applications, interested readers may refer to selected review articles (Inda et al., 2019; Aggarwal et al., 2020).

### 4.1 Systems biology approach to understanding gut commensal biology

An *a priori* understanding of the cellular systems precedes their rational design and engineering. In the case of gut commensals, factors such as the genetic determinants of strain fitness within the intestinal niche provide important engineering considerations for *in vivo* applications. However, conventional approaches to characterize genetic functions, such as gene knockout, are highly effective but limited in scale and require considerable labor and resources (Baba et al., 2006). Therefore, the vast majority of genetic elements in species belong to hypothetical genes that lack validation in literature, even in model gut commensal species.

Attempts to functionally characterize the genetic functions of gut commensal have accelerated using high-throughput screening systems. These include large-scale expression libraries for gain-of-function analyses (Yaung et al., 2015), genome-wide transposon mutagenesis and sequencing (Tn-Seq) (Goodman et al., 2009; Wu et al., 2015; Liu et al., 2021), and CRISPR interference screening platforms for loss-of-function studies (Shin et al., 2023). Such high-throughput sequencing methodologies are an effective means to build and validate novel hypotheses on genotype-phenotype relationships and streamline the investigation of genotype-phenotype relationships in a context-specific manner. For example, a mutant population of *B. thetaiotaomicron* containing a 3.5 × 10^4^ transposon-insertion mutant library that influences 78% of the predicted coding regions at 5.5 insertions/kb resolution was used to survey the essentiality of *B. thetaiotaomicron* genome (Goodman et al., 2009). Transposon insertion sequencing (INSeq) performed *in vitro* and *in vivo* revealed the context-specific nature of gene essentiality, where enriched gene groups differed in functional categories that were a direct reflection of nutrition-replete or -deplete *in vitro* environments. Notably, INSeq also identified genetic determinants of *in vivo* fitness, including the gene cluster BT1957-49 associated with Vitamin B_12_ synthesis and utilization, in which *B. thetaiotaomicron* mobilizes in response to changes in microbiota composition *in vivo* (Goodman et al., 2009). Similarly, specific phenotypes for most of the 516 *B. thetaiotaomicron* genes associated with carbon metabolism and chemical resistance systems were newly characterized via Tn-Seq analysis across a panel of different carbon sources and stressors (Liu et al., 2021).

High-throughput screening of genome-wide transcriptome changes, such as transcriptome sequencing (RNA-Seq), is a common strategy for analyzing condition-specific changes in mRNA transcript abundance. Variations in transcriptome sequencing technologies allow the characterization of distinct transcriptome landscapes across species that reflect unique ecological features and provide an important system-level inference that translates into engineering applications. For example, investigation of *B. thetaiotaomicron* transcriptome response in the presence of sub-components of porcine mucin, a glycoprotein that makes up the mucus lining of the mammalian intestine, has revealed a synchronized action of its PULs along with the regulatory extracytoplasmic sigma factors (ECF-σ). The deletion of genes that encode ECF-σs embedded within *O*-glycan-responsive PULs led to a significant *in vivo* deficit, elucidating yet another essential genetic determinant of *in vivo* fitness, along with the regulatory elements that impart transcriptional control (Martens et al., 2008). Transcriptome analysis has also identified two hybrid TCSs, BT3334 and BT0267, that activate cognate promoters in response to specific carbon substrates (Martens et al., 2008; Martens et al., 2011), which have been repurposed as inducible promoter bioparts in *B. thetaiotaomicron* (Mimee et al., 2015).

More recently, exploration of the primary transcriptome landscape of *B. thetaiotaomicron* genome-wide led to the discovery of more than 4 × 10^3^ transcription start sites corresponding to putative promoters with conserved motifs, conserved ribosome binding sites (RBSs) in the downstream vicinity, and putative riboswitches (Ryan et al., 2020). This discovery has important implications in synthetic biology due to the fact that *Bacteroides* retain a unique σ^70^-like transcription factor σ^ABfr^ that recognizes an unconventional −33/−7 consensus motif (TTTG/TAnnTTTG), and different RBS strength rules that limit the use of standard synthetic biology tools (Mastropaolo et al., 2009). Hence, they add greatly to the pool of promoter and RBS bioparts for *Bacteroides* (Ryan et al., 2020). In addition, differential RNA sequencing readouts provide important inferences regarding the presence of noncoding *cis*-regulatory elements such as riboswitches, many of which assume important regulatory roles but are often omitted from standard genome annotations (Sharma et al., 2010). It is worth highlighting that GibS, a small RNA in *B. thetaiotaomicron* that is strongly induced in the presence of the mucin-constituent amino sugar, *N-*acetyl-D-glucosamine. Upon induction, GibS upregulated the expression of simple sugar metabolic enzymes and downregulated the expression of glucan-branching enzymes, providing evidence for *cis*-regulatory carbon catabolite repression in *B. thetaiotaomicron* (Ryan et al., 2020). As non-coding RNA domains are being harnessed as emerging gene expression machinery in synthetic biology, the accumulation of relevant datasets is expected to streamline novel biopart discovery, especially in non-model organisms, for which validated genetic parts are limited.

### 4.2 Engineering gut commensal-based LBTs

Gut commensals equipped with different features in synthetic biology have been shown to be viable means for therapeutic payload delivery. For example, pioneering research groups engineered *Bacteroides ovatus* to produce anti-inflammatory peptides such as murine interleukin-2 (MuIL2), human keratinocyte growth factor-2 (hKGF-2), and human transforming growth factor-β (hTGF-β) using a xylanase-inducible promoter in pre-clinical models of gut inflammation (Table 2) (Farrar et al., 2005; Hamady et al., 2010; Hamady et al., 2011). The robust heterologous expression system of *Bacteroides* species is often used as a “surrogate cell” to produce potent proteins of intractable commensal origin in the gut for the evaluation of host physiology. For example, a gene encoding tryptophan decarboxylase in the native gut resident *R. gnavus* was heterologously expressed in *B. thetaiotaomicron* to assess the biological implications of this relatively rare pathway that converts tryptophan into tryptamine *in vivo* setting. Gnotobiotic mice colonized with tryptamine-producing *B. thetaiotaomicron* (TrpD ^+^) showed an increase in colonic secretion and a shortened gut transit time (Bhattarai et al., 2018). In a follow-up study, the introduction of *B. thetaiotaomicron* TrpD^+^ decreased weight loss in a colitis mouse model, primarily through tryptamine-induced activation of host serotonin receptor 4 (5-HT4R), which promoted goblet cell differentiation (Bhattarai et al., 2020). Similarly, to evaluate the impact of microbial bile acid metabolism in the colon, genes from *Ruminococcus gnavus* encoding a subset of the 3β-hydroxydeoxycholic acid (isoDCA) biosynthetic pathway, hydroxysteroid dehydrogenases, were heterologously expressed in model *Bacteroides* species (Campbell et al., 2020). Synthetic bile acid metabolism was performed by co-culturing engineered *Bacteroides* strains with *C. scindens*, which encodes the remaining subset of the isoDCA pathway. The introduction of synthetic consortia into the mouse model increased the colonic isoDCA concentration, with a concomitant elevation in the levels of colonic CD4^+^ regulatory T cells (Table 1).

Another avenue of strain engineering is based on a mathematical approach, first to understand biological phenotypes, and second to simulate optimal strain engineering designs that yield the desired outcomes. As mentioned in Section 2, *in silico* simulations using the GEM provide important inferences regarding the implicit dynamics of metabolic interactions within or between the host and microbes. For example, the first GEM reconstruction of *B. thetaiotaomicron i*AH991 was built with the extension of a mouse GEM compartment to simulate host-microbe interactions in response to different dietary regimes. While the multi-species GEM captured metabolite exchanges that were comparable with published metabolomics data, a stand-alone simulation of *i*AH991 for *B. thetaiotaomicron* monoculture phenotypes was incongruent with the experimental values (Heinken et al., 2013). Accumulating knowledge on gut commensal genetics and biochemistry has streamlined the reconstruction of strain-specific GEMs with improved prediction accuracy. Nearly a decade after its reconstruction, *i*AH991 was revisited by incorporating up-to-date strain-specific data and underwent extensive curation of model properties, resulting in an expanded GEM *i*KS1119 that showed remarkably improved *in silico* prediction accuracy (Kim et al., 2021). Subsequently, GEM-guided metabolic engineering was applied to *B. thetaiotaomicron* to produce non-native butyrate, a short-chain fatty acid (SCFA) with proposed immunomodulatory properties. While the strain failed to produce butyrate in the wild-type background, a round of metabolic engineering informed by the Optknock simulation redirected the cellular metabolic flux toward non-native butyrate biosynthesis, albeit at a much lower concentration than the native SCFA products (Kim et al., 2021).

Taken together, these studies demonstrate that prominent gut commensal species can be engineered to functionally express exogenous compounds at a sufficient dosage to elicit the desired changes *in vivo*. Furthermore, the development of *in silico* simulation platforms aids in the rational design and engineering to optimize the production of non-native metabolites. Leveraging this capacity, Novome Biotechnologies Inc. developed a *B. thetaiotaomicron*-based proprietary strain, NB1000S, designed to degrade oxalate for the treatment of enteric hyperoxaluria, and a porphyrin-based prebiotic, NB 2000P, that facilitates engraftment and tunes *in vivo* abundance of NB1000S. The combination product NOV-001 yielded promising results in phase 1 clinical trial assessing safety, tolerability, and strain colonization pharmacodynamics (NCT04909723), paving the way toward the clinical use of commensal-based LBTs that have hitherto been prospective.

## 5 Safety considerations

However, safety concerns regarding the use of genetically engineered bacteria as therapeutic agents remain unresolved. The primary concern is the dissemination of genetically modified bacteria into the environment, posing potential risks, such as horizontal gene transfer (HGT) of artificial genetic elements into native ecosystems. In addition, aberrant behaviors of chassis strains such as *B. thetaiotaomicron* in the expansion of enteric pathogens in genetically-predisposed hosts (Curtis et al., 2014) and invoking pro-inflammatory responses (Brown et al., 2019) call for an effective measure to contain the bioactivity of engineered microbes in a controllable manner. As part of the biocontainment strategy, LBTs are rendered auxotrophic by deleting biosynthetic genes associated with cell survival. In the Synlogic LBT series, *dapA* encoding 4-hydroxy-tetrahydrodipicolinate synthase was inactivated and required exogenous diaminopimelate (DAP) supplementation for survival (Table 2). Other proactive options involve the use of genetic circuits that trigger an actuator response, such as self-lysis effectors, inducer treatment, or exposure to the environment (Chan et al., 2016). Similarly, a “single-use” LBT in which a lysis factor Lysis E7 was integrated into the actuator to induce programmed lysis of the chassis, thereby facilitating the dissemination of anti-pathogenic effector upon sensing pathogen-specific molecule (Table 2) (Saeidi et al., 2011; Hwang et al., 2017). More sophisticated containment strategies are being developed, such as CRISPRi-assisted maintenance of synthetic auxotrophy (article deposited in bioRxiv, refer to Lai et al., 2022 for more details) (Lai et al., 2022). With novel progress in synthetic biology, we expect to see creative endeavors to achieve safer and more efficient LBT biocontainment.

## 6 Conclusion and perspectives

The rapid progress in synthetic biology, accompanied by efforts to decipher complex biology, has culminated in the ability to harness the native gut microbiota to our benefit, which has unveiled a host of novel therapeutic modalities. First, the existing paradigm of microbiome-based therapeutics explores the untapped reservoir of the genetic and biochemical repertoire of the native gut flora, which has been demonstrated to be efficacious, as exemplified by the recent success of VOWST and Rebyota (Adolfsen et al., 2021; Sims et al., 2023), and many more that await clinical assessment.

This marks a promising advancement in the once-controversial fecal microbiota transplant (FMT) therapy, which raised safety flags and was haunted by the weak reproducibility of its therapeutic efficacy. The rational design of synthetic microbial consortia based on a systems biology-driven understanding can selectively screen candidate microbial species and their combinations for maximum therapeutic potency (Tanoue et al., 2019; Campbell et al., 2020). Potentially, the capacity to simulate xenobiotic metabolism in the context of microbiome metagenomics that can account for inter-personal variations (Heinken et al., 2023), can be fully harnessed in challenging areas such as immunocompromised pediatric hosts by informing the most effective therapeutic effects based on predictive analysis of host-specific metabolic features (Flerlage et al., 2020). To this end, the implementation of iterative cycles of design-build-test-learn (DBTL) to systematically assess the outcome of the built designs, learn the system function, and install novel features to obtain an optimally functioning microbiome therapeutic product (Lawson et al., 2019).

Specific avenues for microbiome therapeutics require genetic engineering to augment strain performance and endow nonnative functionalities. In addition to direct microbe-to-host delivery, LBTs equipped with reliable biosensors can now execute diagnoses and site-specific dose-dependent payload delivery (production) *in vivo*. One prospect of the LBT lies in its ability to administer the correct drug dosage at the right time and place, which is expected to significantly improve treatment efficiency while minimizing side effects that are not uncommon in current therapeutic regimens (Inda et al., 2019). Furthermore, the emerging utility of gut commensals as chassis strains is expected to overcome several obstacles in probiotic-based *in vivo* therapeutic applications such as engraftment efficiency, rapid clearance, and low cell density. The rapid pace of development in the synthetic biology of gut commensals has culminated in NOV-001, the first gut commensal-based LBT that recently passed a phase 1 clinical trial (NCT04909723), paving the way toward real-life applications of commensal-based therapeutics that have long remained prospective. However, a recent report showed a distinct approach to circumvent the existing hurdles in probiotic (*E. coli*)-based LBTs to isolate and engineer native gut-resident *E. coli* which engrafts perpetually upon reintroduction (Russell et al., 2022). This is an interesting development in native gut commensal engineering and is worth considering in future LBT applications.

Living cells constantly undergo evolution, similar to that of LBTs. SYNB1618, the original Phe-degrading LBT that passed a phase 1 trial (NCT03516487), was upgraded using a catalytically superior mutant PAL enzyme discovered through high-throughput screening (Adolfsen et al., 2021). In another independent study, the transgene expression architecture of SYNB1618 was remodeled using an AutoCAD (Cello)-assisted genetic circuit design that reduced cellular burden (Triassi et al., 2023). The progress in the Phe-degrading LBT series implies that the ‘rooms for development’ in the existing chassis may be considerably wider than we previously thought. Machine learning algorithms expedite the interpretation of large agglomerates of high-throughput datasets, facilitating the discovery of hidden layers of regulation that can be translated into pragmatic applications (Sastry et al., 2019; Rychel et al., 2021). High-throughput automated screening platforms combine a wealth of (un)validated bioparts to streamline the exploration of solution spaces for optimal strain function (Yu et al., 2023). Unparalleled advances in systems and synthetic biology will accelerate the pace of chassis development and expedite the evolution of LBT performance.
